# Monosodium urate crystals reduce osteocyte viability and indirectly promote a shift in osteocyte function towards a proinflammatory and proresorptive state

**DOI:** 10.1186/s13075-018-1704-y

**Published:** 2018-09-10

**Authors:** Ashika Chhana, Bregina Pool, Karen E. Callon, Mei Lin Tay, David Musson, Dorit Naot, Geraldine McCarthy, Susan McGlashan, Jillian Cornish, Nicola Dalbeth

**Affiliations:** 1 Department of Medicine, Bone & Joint Research Group, University of Auckland, Auckland, New Zealand; 2 Department of Rheumatology, Mater Misericordiae University Hospital, Dublin, Ireland; 3 Department of Anatomy and Medical Imaging, University of Auckland, Auckland, New Zealand; 4 Department of Medicine, Faculty of Medical and Health Sciences, University of Auckland, 85 Park Rd, Grafton, Auckland, New Zealand

**Keywords:** Gout, Osteocyte, Inflammation, Urate, Bone erosion

## Abstract

**Background:**

Bone erosion is a frequent complication of gout and is strongly associated with tophi, which are lesions comprising inflammatory cells surrounding collections of monosodium urate (MSU) crystals. Osteocytes are important cellular mediators of bone remodeling. The aim of this study was to investigate the direct effects of MSU crystals and indirect effects of MSU crystal-induced inflammation on osteocytes.

**Methods:**

For direct assays, MSU crystals were added to MLO-Y4 osteocyte cell line cultures or primary mouse osteocyte cultures. For indirect assays, the RAW264.7 macrophage cell line was cultured with or without MSU crystals, and conditioned medium from these cultures was added to MLO-Y4 cells. MLO-Y4 cell viability was assessed using alamarBlue® and LIVE/DEAD® assays, and MLO-Y4 cell gene expression and protein expression were assessed by real-time polymerase chain reaction (PCR) and enzyme-linked immunosorbent assay (ELISA), respectively. Histological analysis was used to examine the relationship between MSU crystals, inflammatory cells, and osteocytes in human joints affected by tophaceous gout.

**Results:**

In direct assays, MSU crystals reduced MLO-Y4 cell and primary mouse osteocyte viability but did not alter MLO-Y4 cell gene expression. In contrast, conditioned medium from MSU crystal-stimulated RAW264.7 macrophages did not affect MLO-Y4 cell viability but significantly increased MLO-Y4 cell expression of osteocyte-related factors including E11, connexin 43, and RANKL, and inflammatory mediators such as interleukin (IL)-6, IL-11, tumor necrosis factor (TNF)-α and cyclooxygenase-2 (COX-2). Inhibition of COX-2 in MLO-Y4 cells significantly reduced the indirect effects of MSU crystals. In histological analysis, CD68^+^ macrophages and MSU crystals were identified in close proximity to osteocytes within bone. COX-2 expression was also observed in tophaceous joint samples.

**Conclusions:**

MSU crystals directly inhibit osteocyte viability and, through interactions with macrophages, indirectly promote a shift in osteocyte function that favors bone resorption and inflammation. These interactions may contribute to disordered bone remodeling in gout.

**Electronic supplementary material:**

The online version of this article (10.1186/s13075-018-1704-y) contains supplementary material, which is available to authorized users.

## Background

Bone erosion is a common complication of tophaceous gout [1]. Tophi are ordered structures containing inflammatory cells and tissue surrounding collections of monosodium urate (MSU) crystals [2]. Both MSU crystals and the soft tissue components of the tophus are strongly and independently associated with bone erosion in gout [3].

Joints affected by tophaceous gout show evidence of disordered bone remodeling, with increased osteoclast-mediated bone resorption [4, 5] and impaired osteoblast-mediated bone formation [6]. Osteocytes are the most abundant cell type found within bone and are important regulators of bone remodeling, controlling both bone resorption and bone formation [7, 8]. Embedded within the mineralized matrix, osteocytes communicate with each other and other cells on the bone surface through dendrites. Osteocytes are also a source of soluble factors that can target local and distant tissues [9]. We have previously reported that osteocyte-derived soluble factors mediate the relationship between tophus and erosion in gout [10], suggesting that osteocytes may contribute to the development of bone erosion in gout. The aim of this study was to investigate the direct effects of MSU crystals and indirect effects of MSU crystal-induced inflammation on osteocyte viability and function.

## Methods

### Ethical approvals

Human sample collection was approved by the Northern Regional Ethics Committee and all participants provided written informed consent. Protocols involving animals were approved by the University of Auckland Animal Ethics Committee. Use of human cadaveric tissue was in accordance with the New Zealand Human Tissue Act 2008.

### Cell culture

MLO-Y4 cells (a kind gift from Professor Lynda Bonewald, Indiana University) were cultured in α-minimum essential medium (MEM) containing l-glutamine and nucleosides (Gibco, Life Technologies, Thermo Fisher Scientific, Waltham, USA) and supplemented with 2.5% heat-inactivated fetal bovine serum (FBS; Gibco) and 2.5% heat-inactivated newborn calf serum (Hyclone, GE Healthcare Life Sciences, Logan, USA). The MLO-Y4 cell line is widely used for in-vitro studies of osteocytes; these cells have similar properties to primary osteocytes with high expression of osteocalcin and connexin 43. MLO-Y4 cells also have dendritic processes and can communicate via gap junctions, similar to primary osteocytes [11, 12].

Primary mouse osteocytes were isolated from the long bones of 6-week-old C57BL/6 male mice using a modified method of Stern et al. [13]. The long bones (femur, tibia, and humerus) were harvested, the epiphyses removed, and bone marrow flushed out with α-MEM. Bones were then cut into small pieces and sequentially digested nine times using 300 U/mL collagenase (Sigma-Aldrich, St. Louis, USA; digests 1–3, 5, 7, and 9; 25 min per digest) and 5 mM EDTA (Sigma-Aldrich; digests 4, 6, and 8; 40 min per digest) at 37 °C. Bone chips were washed with Hank’s balanced salt solution (Gibco) between digests. Osteocyte-like cells were grown out from the digested bone chips onto collagen-coated surfaces with the same medium that was used for MLO-Y4 cells.

For all experiments, MLO-Y4 cells or primary mouse osteocytes were seeded in three-dimensional (3D) collagen gels in 24-well plates (1 × 10^4^ cells/50 μL gel; 1 gel/well) as previously described [14]. Briefly, rat collagen type I (Corning Inc., Corning, USA) was neutralized with 1 M NaOH and diluted to a final concentration of 3 mg/mL. Cells were seeded in 50 μL collagen gels and allowed to set at 37 °C for 1 h prior to the addition of 1 mL culture medium. Cells were cultured in the collagen gels for 4 days (MLO-Y4 cells) or 24 h (primary mouse osteocytes) before the media were replaced and experiments commenced.

The RAW264.7 macrophage cell line (ATCC, Manassas, USA) was maintained in Iscove’s modified Dulbecco’s medium supplemented with 1 mM l-glutamine (both from Sigma-Aldrich) and 10% FBS.

### MSU crystal synthesis

Endotoxin-free MSU crystals were prepared by recrystallization from uric acid as previously described [15].

### Preparation of conditioned medium from RAW264.7 macrophages

RAW264.7 cells were seeded in 24-well plates at 1 × 10^6^ cells/well. The following day, the medium was changed to α-MEM containing l-glutamine and nucleosides, supplemented with 2.5% heat-inactivated FBS and 2.5% heat-inactivated newborn calf serum, and 0.5 mg/mL MSU crystals were added for 24 h. Conditioned medium was then harvested and filtered using a 0.2-μm filter to remove any residual MSU crystals. The absence of MSU crystals was confirmed by polarizing light microscopy. Control conditioned medium from RAW264.7 macrophages alone (no added MSU crystals) was also prepared at the same time. There were up to 18 wells in each treatment group.

### alamarBlue® assay for cell viability

For direct assays, various concentrations of MSU crystals were added to MLO-Y4 cells or primary mouse osteocytes for 24 h. Cells were then washed to remove MSU crystals and alamarBlue® reagent (Life Technologies) was added (5% final concentration in a well) for 6 h at 37 °C. At the same time, 1 U/mL uricase (Sigma-Aldrich) was added to remove residual MSU crystals to prevent interference with the assay [6]. Cell viability was assessed both 24 and 48 h after the addition of MSU crystals by measuring fluorescence (excitation 540 nm; emission 630 nm) using a Synergy 2 multidetection microplate reader (BioTek Instruments Inc., Winooski, VT). In separate experiments, calcium pyrophosphate dihydrate (CPPD) crystals (Integrated Sciences, Sydney, Australia), basic calcium phosphate (BCP) crystals (synthesized as described previously [16]) and aluminum particulates (Sigma-Aldrich) were also added to MLO-Y4 cells for viability assays.

For indirect assays, 5%, 20%, or 40% control conditioned medium or MSU crystal-stimulated conditioned medium (from the RAW264.7 macrophage assays) was added to MLO-Y4 cells for 24 h. Cells were then washed, and viability assessed using alamarBlue® as above. There were up to six wells in each treatment group for all experiments.

### LIVE/DEAD® assay for cell viability

MSU crystals (0.1 or 0.3 mg/mL) were added to MLO-Y4 cells for 24 h. Cells were washed, and crystals completely removed. Cells were then stained with calcein-AM (live cells) and ethidium homodimer-1 (dead cells) using the LIVE/DEAD® Viability/Cytotoxicity Kit (Life Technologies), either 24 or 48 h after the addition of MSU crystals. Fluorescence microscopy was used to take 10 paired images of stained cells (live and dead) within three separate layers of the collagen gel (top, middle, and bottom). ImageJ software (https://imagej.nih.gov/ij/) was used to count the number of living or dead cells in each gel layer and the percentage of dead cells was calculated.

### Gene and protein expression assays

For direct assays, 0.1 mg/mL MSU crystals were added to MLO-Y4 cells for 0, 1, 6, and 24 h. For indirect assays, 40% RAW264.7 conditioned medium (control or MSU crystal-stimulated) was added to MLO-Y4 cells for 0, 1, 6, and 24 h. MLO-Y4 cells were harvested for gene expression analysis, and MLO-Y4 cell supernatants and RAW264.7 macrophage conditioned medium preparations were harvested for secreted protein analysis. For each experiment, there were 6–12 wells in each treatment group.

#### Quantitative real-time polymerase chain reaction (PCR)

Purification of total cellular RNA, synthesis of cDNA, and real-time PCR was performed as previously described [14]. 18S rRNA endogenous control was used to correct for variations in cell numbers between samples. The ΔΔCt method was used to calculate the relative levels of gene expression, using day 0 or control cell (MLO-Y4 cells alone) expression levels as a control.

#### Protein quantification

Protein levels of tumor necrosis factor (TNF)-α, interleukin (IL)-6, IL-1β, soluble receptor activator of nuclear factor kappa-B ligand (RANKL), and osteoprotegerin (OPG) in RAW264.7 macrophage conditioned medium preparations and MLO-Y4 supernatants was determined by enzyme-linked immunosorbent assay (ELISA) (R&D Systems, Minneapolis, USA). Prostaglandin E_2_ (PGE_2_) was measured in conditioned media and supernatant samples as a measure of cyclooxygenase-2 (COX-2) enzyme activity using the PGE_2_ EIA Kit (Cayman Chemical, Ann Arbor, USA).

#### TNF-α and COX-2 inhibition experiments

For TNF-α blocking experiments, 5 μg/mL TNF-α neutralizing antibody (monoclonal rat IgG1, clone MP6-XT22) or IgG1 isotype control (both from R&D Systems) was added to MLO-Y4 cells for 1 h prior to the addition of RAW264.7 macrophage conditioned medium for 24 h. The concentration of TNF-α neutralizing antibody was chosen based on optimization experiments whereby three different concentrations (0.5, 2.5, and 5 μg/mL) of neutralizing antibody was added to MLO-Y4 cells prior to the addition of 4 ng/mL TNF-α for 24 h. The addition of 5 μg/mL TNF-α neutralizing antibody suppressed TNF-α-induced expression of COX-2, IL-11, and RANKL genes by MLO-Y4 cells (data not shown).

For COX-2 blocking experiments, 1 μM COX-2-specific inhibitor (SC-236, Sigma-Aldrich) was added to MLO-Y4 cells for 1 h prior to the addition of RAW264.7 macrophage conditioned medium for 24 h.

### Histology of joint samples affected by gout

Human joint samples (two each from finger proximal and distal interphalangeal joints, and one each from the knee, mid-foot, and a big toe interphalangeal joint) were obtained from two patients with gout undergoing orthopedic surgery and three cadaveric donors with microscopically proven gout. Cadaveric samples were transferred to 70% ethanol immediately after collection and all samples were demineralized at room temperature in 10% formic acid for 1 week prior to paraffin embedding. Slides were prepared and stained with toluidine blue as previously described [17] or used for immunohistochemistry. The spatial relationship between osteocytes, macrophages, and MSU crystals in joint samples was examined using polarizing light microscopy. Immunohistochemistry was used to identify CD68^+^ macrophage cells and COX-2 expression.

#### Immunohistochemistry for CD68 and COX-2

Sections were dewaxed for 12 min in Safsolvent (Ajax Finechem Pty Ltd., Melbourne, Australia) and rehydrated through graded ethanol solutions for 5 min each. Once hydrated, sections were immersed in 0.5% pepsin for 14 min at 37 °C (CD68) or pH 9.0 Dako Target Retrieval Solution (Produktionsvej, Denmark) for 20 min at 96 °C (COX-2). To block endogenous peroxidase activity, sections were incubated in Dual Endogenous Enzyme-Blocking Reagent (Dako, Produktionsvej, Denmark) for 20 min (CD68) or 3% hydrogen peroxide in methanol for 15 min (COX-2). To block nonspecific binding, sections were incubated in 10% goat serum for 30 min (CD68) or 3% bovine serum albumin (MP Biomedicals New Zealand, Auckland, New Zealand) for 30 min (COX-2). Sections were incubated with primary antibody (1:200 dilution of anti-human CD68 clone PG-M1, Dako; 1:100 dilution of anti-human COX-2, clone SP21, Thermo Fisher Scientific) at 4 °C overnight. After washing with phosphate-buffered saline (PBS), slides were incubated with the Dako Dual link system peroxidase secondary antibody for 30 min (CD68) or 2 h (COX-2). After further washing, the Impact DAB Substrate Kit (Vector Laboratories, Burlingame, CA) was used to detect staining according to the manufacturer’s instructions. Slides were briefly counterstained with Hematoxylin QS counter stain (Vector Laboratories) and dehydrated through graded ethanol solutions and xylol. Slides were mounted with DPX (BDH, Poole, UK) and analyzed by light microscopy.

### Statistical analysis

Data were analyzed using SAS Software (SAS Institute, Cary, USA) and GraphPad Prism Software (v7, GraphPad Software, San Diego, USA). For all experiments, data were pooled from three to five biological repeats. Data were analyzed using one-way or two-way analysis of variance (ANOVA) with post-hoc Dunnett’s or Sidak’s multiple comparison tests in the case of more than two groups, or by two-tailed paired *t* test in the case of two groups.

## Results

### MSU crystals directly reduce MLO-Y4 cell and primary mouse osteocyte cell viability over time

**Fig. 1:**
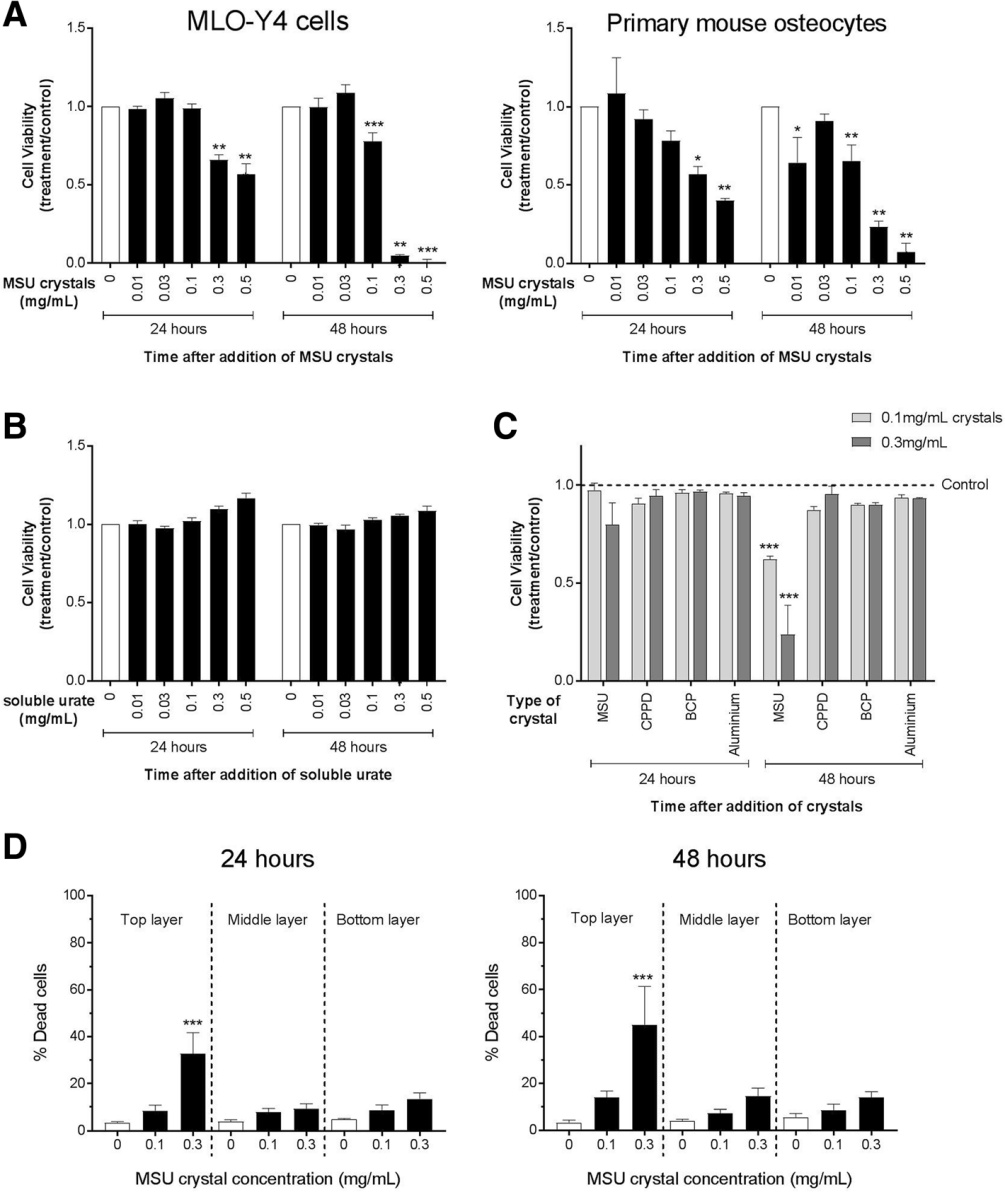
The direct effects of MSU crystals on osteocyte viability. The alamarBlue® assay was used to determine the viability of **a** MLO-Y4 cells and primary mouse osteocytes cultured with monosodium urate (MSU) crystals for 24 h, **b** MLO-Y4 cells cultured with soluble urate for 24 h, and **c** MLO-Y4 cells cultured with different types of crystals for 24 h. Viability was assessed 24 and 48 h after the addition of crystals or soluble urate. Data shown are pooled from three to four biological repeats and are presented as mean (SEM); by two-way ANOVA **a** *P*_Interaction_ < 0.0001 for MLO-Y4 cells, *P*_Interaction_ = 0.026 for primary mouse osteocytes, **b** *P*_Interaction_ = 0.24, and **c** *P*_Interaction_ = 0.057 at 24 h, *P*_Interaction_ < 0.0001 at 48 h; with post-hoc Dunnett’s test *p < 0.05, **p < 0.01, and ***p < 0.001 versus control (no crystals or soluble urate) at that time point. **d** The LIVE/DEAD® assay was used to determine the percentage of dead MLO-Y4 cells within three separate layers of the collagen gel following culture with MSU crystals for 24 h or 48 h. Data shown are pooled from four biological repeats and are presented as mean (SEM); one-way ANOVA *p* < 0.0001 at 24 h, *p* = 0.004 at 48 h; with post-hoc Sidak’s test ***p < 0.001 versus control (no MSU crystals) for each layer of the gel. BCP basic calcium phosphate, CPPD calcium pyrophosphate dehydrate

The higher concentrations of MSU crystals (0.3–0.5 mg/mL) reduced the viability of MLO-Y4 cells and primary mouse osteocytes after 24 h as assessed by alamarBlue® assays, with a further reduction in viability observed at the 48 h time point (Fig. 1a). The inhibitory effect was specific to MSU crystals, since soluble urate at the same concentrations (Fig. 1b) and other types of crystals (CPPD, BCP, aluminum) did not reduce MLO-Y4 cell viability (Fig. 1c). The effects on MLO-Y4 cell viability were not altered with different MSU crystal lengths (Additional file 1: Figure S1).

To assess whether MLO-Y4 cell death induced by MSU crystals was consistent throughout the 3D collagen gel, LIVE/DEAD® assays were performed and MLO-Y4 cell death in the top, middle, and bottom layers of the collagen gel were determined. In these assays, significant MLO-Y4 cell death was observed in the top layer of the gel compared with the middle and bottom layers following culture with 0.3 mg/mL MSU crystals for 24 h or 48 h (Fig. 1d).

### MSU crystals do not directly alter MLO-Y4 cell expression of bone-related or inflammatory genes

**Fig. 2:**
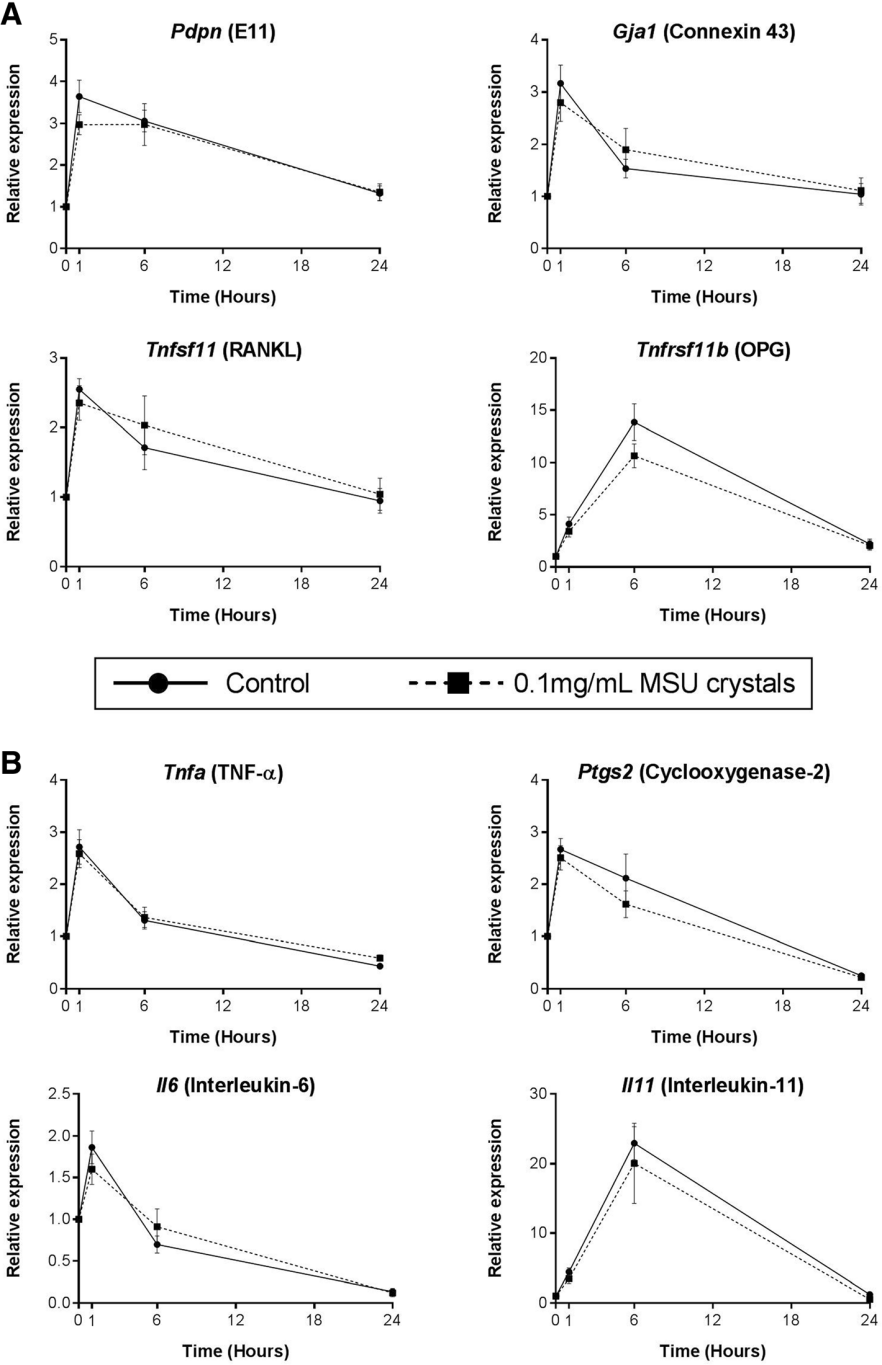
Direct effects of MSU crystals on MLO-Y4 cell expression of bone-related or inflammatory genes. Real-time PCR was used to determine changes in the relative mRNA expression levels of **a** bone-related and **b** inflammatory genes in MLO-Y4 cells, following culture with 0.1 mg/mL monosodium urate (MSU) crystals for 0, 1, 6, and 24 h. Data shown are pooled from three biological repeats and are presented as mean (SEM); two-way ANOVA *P*_Interaction_ > 0.1 for all genes. OPG osteoprotegerin, RANKL receptor activator of nuclear factor kappa-B ligand, TNF tumor necrosis factor

Real-time PCR was used to determine changes in gene expression in MLO-Y4 cells cultured with MSU crystals for 1, 6, and 24 h. MSU crystals alone did not alter the expression of bone-related genes, including E11 (*Pdpn*), connexin 43 (*Gja1*), RANKL (*Tnfsf11*), or OPG (*Tnfrs11b*) (Fig. 2a), or inflammatory genes, including TNF-α (*Tnfa*), COX-2 (*Ptgs2*), IL-6 (*Il6*), and IL-11 (*Il11*) (Fig. 2b). IL-1β (*Il1b*) was not expressed by MLO-Y4 cells.

### Conditioned medium from MSU crystal-stimulated RAW264.7 macrophages has no effect on MLO-Y4 cell viability

To assess whether soluble factors released by macrophages in response to MSU crystals indirectly affect osteocyte viability, RAW264.7 cells were cultured with or without 0.5 mg/mL MSU crystals for 24 h, and conditioned medium was harvested. The addition of increasing concentrations of control or MSU crystal-stimulated conditioned medium had no effect on MLO-Y4 cell viability as determined by the alamarBlue® assay after 24 and 48 h (Additional file 2: Figure S2).

### Conditioned medium from MSU crystal-stimulated RAW264.7 macrophages alters MLO-Y4 cell expression of bone-related factors and upregulates MLO-Y4 expression of inflammatory mediators

**Fig. 3:**
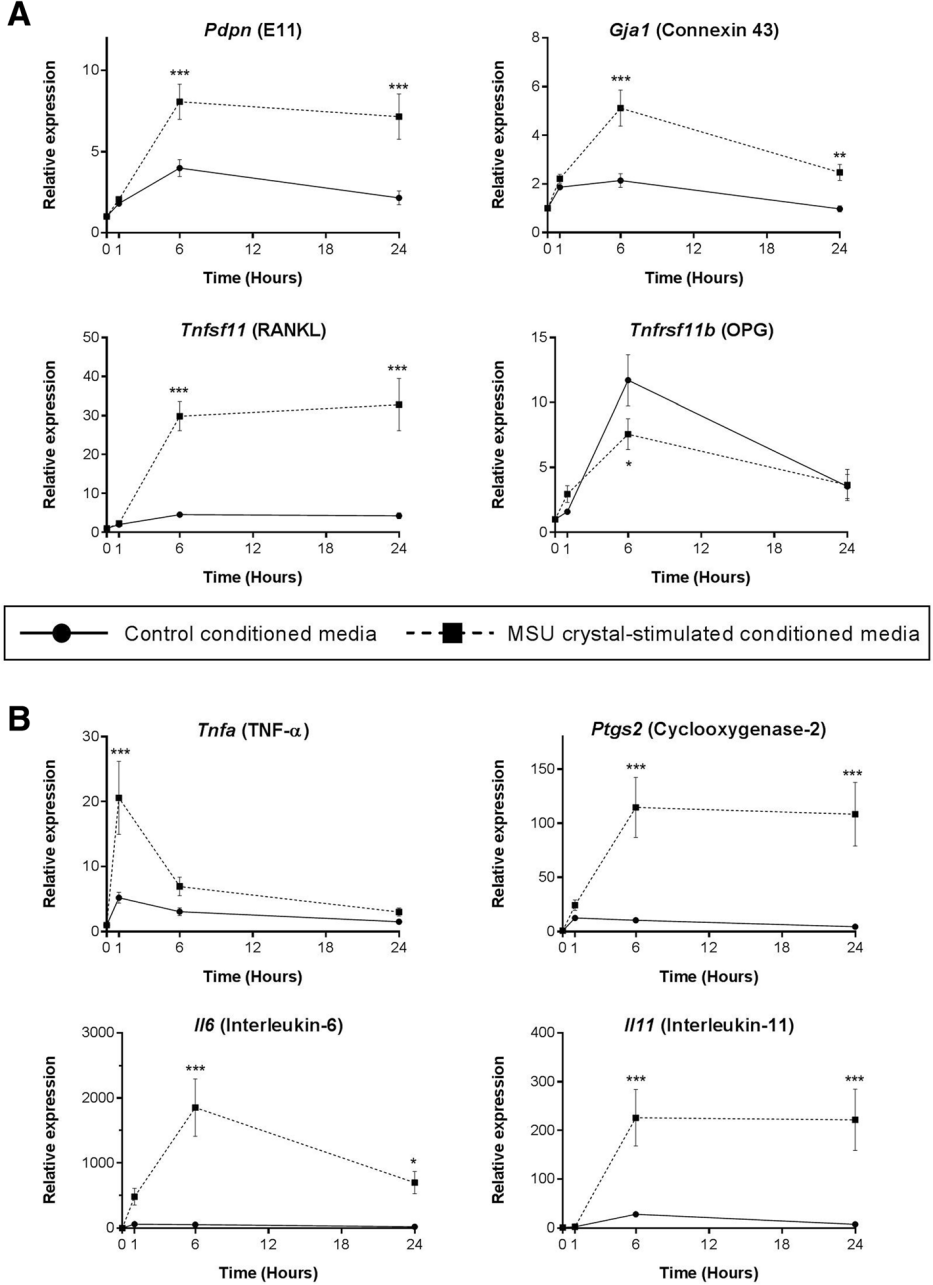
Indirect effects of MSU crystal-stimulated RAW264.7 macrophage conditioned medium on MLO-Y4 cell gene expression. RAW264.7 macrophages were cultured with or without 0.5 mg/mL monosodium urate (MSU) crystals for 24 h for preparation of MSU crystal-stimulated conditioned medium and control conditioned medium, respectively. Conditioned medium preparations were added to MLO-Y4 cells (40% final concentration in a well) for 0, 1, 6, and 24 h. MLO-Y4 cells were harvested and real-time PCR was used to determine changes in the relative mRNA expression levels of **a** bone-related and **b** inflammatory genes. Data shown are pooled from three biological repeats and are presented as mean (SEM); two-way ANOVA *P*_Interaction_ = 0.007 for *Tnfrsf11b*, *P*_Interaction_ = 0.0005 for *Tnfa*, *P*_Interaction_ < 0.0001 for all other genes; with post-hoc Sidak’s test *p < 0.05, **p < 0.01, and ***p < 0.001 versus control conditioned medium at that time point. OPG osteoprotegerin, RANKL receptor activator of nuclear factor kappa-B ligand, TNF tumor necrosis factor

The addition of 40% conditioned medium from MSU crystal-stimulated RAW264.7 macrophages to MLO-Y4 cell cultures led to an approximate two- to fourfold increase in expression of E11 and connexin 43 at the 6 and 24 h time points compared with control conditioned medium. RANKL expression was upregulated approximately sixfold at the 6 and 24 h time points, and OPG expression was reduced at the 6 h time point by approximately twofold (Fig. 3a).

The expression of inflammatory genes by MLO-Y4 cells was also significantly upregulated with the addition of MSU crystal-stimulated conditioned medium, including TNF-α (~ 4-fold) after 1 h, COX-2 (~ 11-fold) after 6 and 24 h, IL-6 after 6 and 24 h (~ 1800-fold and ~ 700-fold, respectively), and IL-11 (~ 200-fold) after 6 and 24 h (Fig. 3b).

**Fig. 4:**
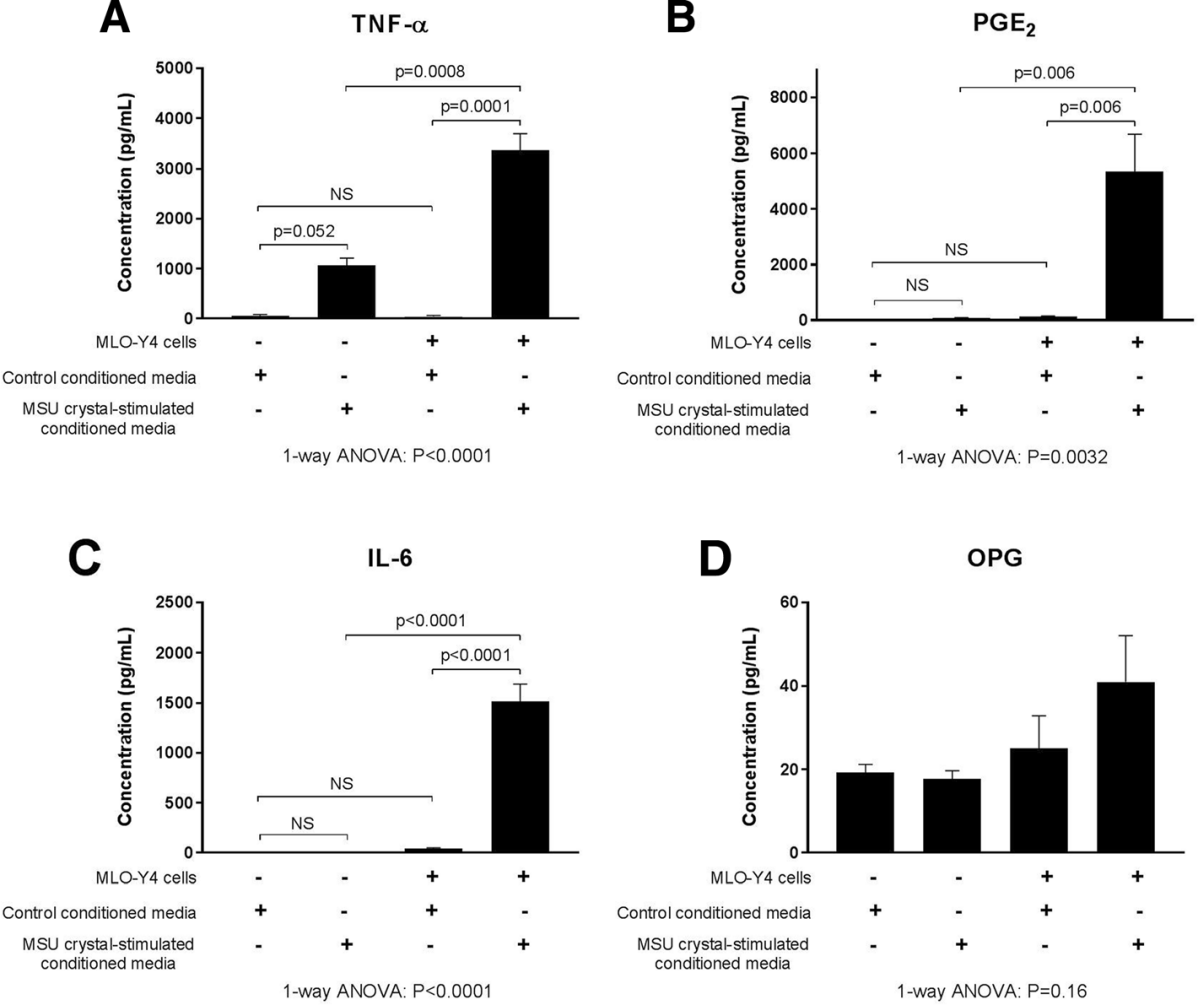
Secretion of proinflammatory mediators by MLO-Y4 cells in response to MSU crystal-stimulated RAW264.7 macrophages. RAW264.7 macrophages were cultured with or without 0.5 mg/mL monosodium urate (MSU) crystals for 24 h for preparation of MSU crystal-stimulated conditioned medium and control conditioned medium, respectively. Conditioned medium preparations were added to MLO-Y4 cells (40% final concentration in a well) for 24 h and supernatants harvested. The concentrations of **a** tumor necrosis factor (TNF)-α, **b** prostaglandin E_2_ (PGE_2_), **c** interleukin (IL)-6, and **d** osteoprotegerin (OPG) protein in the RAW264.7 macrophage conditioned medium samples (control and MSU crystal-stimulated) and the MLO-Y4 cell supernatants were measured by ELISA. Data shown are pooled from three biological repeats and are presented as mean (SEM); one-way analysis of variance (ANOVA) with post-hoc Sidak’s test between groups as indicated. NS no significant difference

To assess changes at the protein level, MLO-Y4 cell supernatants were harvested and protein concentrations were measured in the MLO-Y4 cell supernatants and compared with RAW264.7 macrophage conditioned medium alone (before addition to MLO-Y4 cells). High levels of TNF-α, PGE_2_, and IL-6 protein were secreted by MLO-Y4 cells in response to conditioned medium from RAW264.7 macrophages cultured with MSU crystals (Fig. 4a–c). OPG protein levels were unchanged (Fig. 4d) and soluble RANKL was not detected in any conditioned media or supernatant samples.

### The inflammatory response induced in MLO-Y4 cells by conditioned medium from MSU crystal-stimulated RAW264.7 macrophages is suppressed with inhibition of COX-2

The concentration of candidate inflammatory mediators and bone factors in conditioned medium collected from RAW264.7 macrophages was measured using ELISA. The addition of MSU crystals to RAW264.7 macrophages led to significantly increased secretion of TNF-α protein and PGE_2_ compared with control cells (without MSU crystals) (Additional file 3: Figure S3). There was no change in IL-1β or OPG release, and IL-6 and soluble RANKL protein were undetected in both control conditioned medium and MSU crystal-stimulated conditioned medium (Additional file 3: Figure S3).

**Fig. 5:**
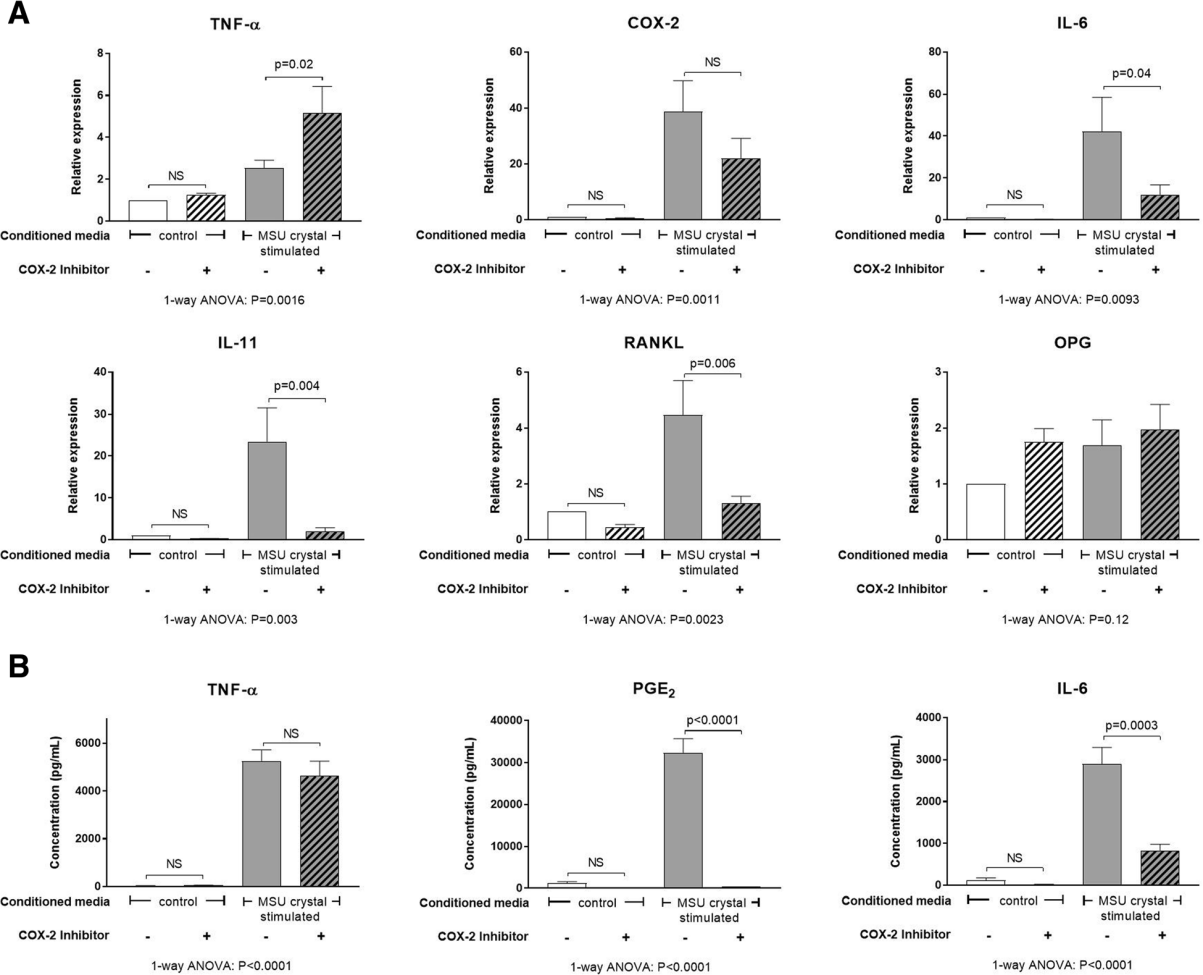
Effects of COX-2 inhibition on MLO-Y4 cell responses to MSU crystal-stimulated RAW264.7 macrophage conditioned medium. RAW264.7 macrophages were cultured with or without 0.5 mg/mL monosodium urate (MSU) crystals for 24 h for preparation of MSU crystal-stimulated conditioned medium and control conditioned medium, respectively. A cyclooxygenase-2 (COX-2)-specific inhibitor (SC-236) was added to MLO-Y4 cells for 1 h prior to the addition of 40% conditioned medium for 24 h. MLO-Y4 cells were then harvested for mRNA gene expression analysis and supernatants harvested for protein quantification. **a** Changes in mRNA expression of inflammatory genes: tumor necrosis factor (TNF)-α, COX-2, interleukin (IL)-6, and IL-11; and bone-related genes: receptor activator of nuclear factor kappa-B ligand (RANKL) and osteoprotegerin (OPG). **b** Changes in TNF-α, prostaglandin E_2_ (PGE_2_), and IL-6 protein levels in MLO-Y4 cell supernatants. Data shown are pooled from five biological repeats and are presented as (SEM); one-way analysis of variance (ANOVA) with post-hoc Sidak’s test between groups as indicated. NS no significant difference

The addition of TNF-α neutralizing antibody did not significantly change the induced expression of COX-2, IL-6, IL-11, and RANKL genes by MLO-Y4 cells following culture with MSU crystal-stimulated conditioned medium (Additional file 4: Figure S4). In contrast, addition of a COX-2 inhibitor to MLO-Y4 cell cultures prior to the addition of MSU crystal-stimulated conditioned medium led to a significant reduction in IL-6, IL-11, and RANKL gene expression (Fig. 5a), and PGE_2_ and IL-6 protein expression (Fig. 5b). TNF-α gene expression was slightly increased following COX-2 inhibition (Fig. 5a); however, there was no difference at the protein level (Fig. 5b). MLO-Y4 cell gene expression of OPG and COX-2 was unchanged (Fig. 5a); however, PGE_2_ levels were significantly decreased with the addition of COX-2 inhibitor (Fig. 5b). Soluble RANKL protein was undetected in the MLO-Y4 cell supernatants.

### MSU crystals and macrophages are observed in close proximity to bone, and COX-2 is also expressed in human joints affected by tophaceous gout

**Fig. 6:**
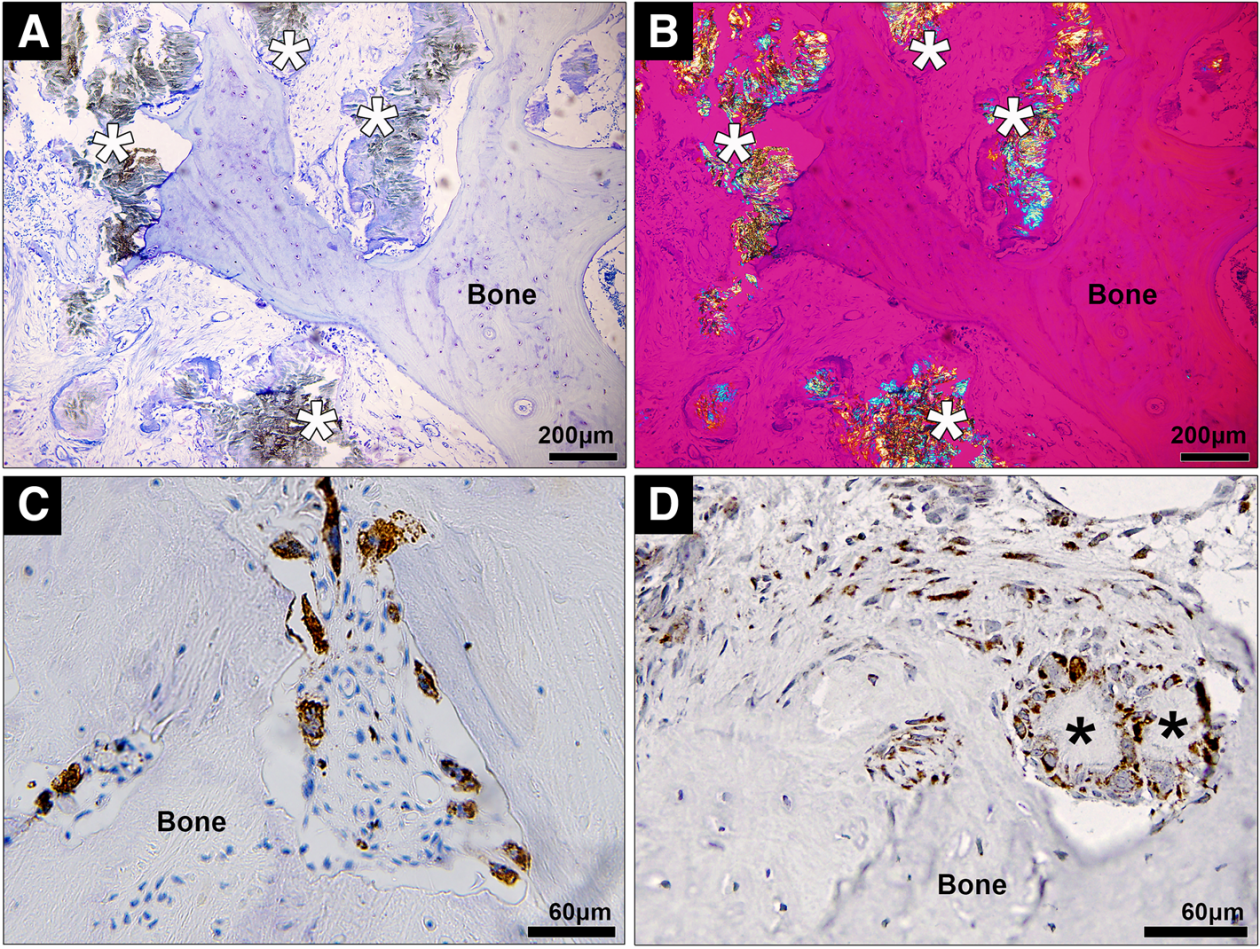
Histological analysis of human joint tissue affected by tophaceous gout. **a,b** Representative photomicrographs of joint samples affected by tophaceous gout, showing both MSU crystals (indicated by asterisks) and associated inflammatory tissue in close proximity to bone (**a**, toluidine blue staining viewed using light microscopy; **b**, viewed using polarizing light microscopy with a red compensator). Immunohistochemistry staining for **c** CD68^+^ cells (macrophages) and **d** COX-2 expression in human joint tissue affected by tophaceous gout

The clinical relevance of our in-vitro results was assessed by examining the relationship between MSU crystals, inflammatory cells, and bone in human joint tissue affected by gout. In joint samples from people with tophaceous gout, collections of MSU crystals were observed both immediately adjacent to the bone (direct contact) and also distant from the bone, separated from the bone surface by a rim of inflammatory tissue (Fig. 6a, b). Multiple CD68^+^ macrophages were identified within tophi adjacent to the bone (Fig. 6c). In addition, COX-2 expression was observed in both mononucleated and multinucleated cells within the corona zone of tophi and in cells close to the bone in joints affected by tophaceous gout (Fig. 6d).

## Discussion

This study shows that MSU crystals have significant inhibitory effects on osteocyte viability, but no direct effect on osteocyte gene expression in vitro. In contrast, in conditioned media experiments, factors released by macrophages in response to MSU crystals have profound effects on the expression profile of osteocytes, with upregulated expression of inflammatory cytokines and mediators, and altered expression of factors involved in bone remodeling. The shift in osteocyte function towards a proinflammatory and proresorptive state is effectively suppressed with COX-2 inhibition.

Our in-vitro assays demonstrate that MSU crystals directly reduce the viability of osteocytes embedded within 3D collagen gels over time. Although the alamarBlue® results showed that direct cell-crystal contact was not necessarily required for this effect, the increased level of cell death observed in the top layer of the gel in the LIVE/DEAD® assays suggests that direct cell-crystal contact may enhance MSU crystal-induced osteocyte death. Osteocyte cell death is associated with increased osteoclastogenesis and loss of bone [18], with dying osteocytes and their neighboring cells thought to send signals which recruit osteoclast precursors to areas of bone damage [19, 20]. In patients with tophaceous gout, large numbers of osteoclasts are present at the bone-tophus interface at the site of bone erosion [4]. Increased osteocyte death in the presence of MSU crystals may further amplify osteoclast precursor cell recruitment, osteoclastogenesis, and bone resorption at these sites.

MSU crystals did not directly alter MLO-Y4 gene expression in our 3D in-vitro cell culture model and, while direct interaction of MSU crystals with cells in the deeper layers of the gels may have been limited, this model is more representative of osteocytes in vivo which are embedded within a 3D matrix. The histology analysis confirmed that, while MSU crystals are in direct contact with surface bone cells, there is often a rim of macrophages and inflammatory tissue observed between MSU crystals and osteocytes within the bone. Interactions between nearby MSU crystals and macrophages and the resulting inflammation may also influence osteocyte regulation of bone remodeling in joints affected by tophaceous gout. The addition of conditioned medium from macrophages cultured with MSU crystals to MLO-Y4 cells led to upregulated expression of genes involved in osteocyte communication (connexin 43 and E11), indicating that the cells may be responding to the external stress [21, 22]. In addition, cytokines and factors known to increase osteoclastogenesis and bone resorption were also upregulated in the MLO-Y4 cell cultures, including TNF-α [23], IL-6 [24], IL-11 [25], and RANKL [26]. COX-2 gene expression was also upregulated in response to MSU crystal-stimulated conditioned medium. Induction of COX-2 leads to the synthesis of prostaglandins, such as PGE_2_. In the same experiments, PGE_2_ levels were significantly increased in response to MSU crystal-stimulated conditioned medium. PGE_2_ has important effects on bone metabolism and can promote both bone resorption and formation [27, 28]. IL-6 and IL-11 are also known to have roles in promoting bone formation under conditions of increased bone turnover [29, 30]. Of note, pathological new bone formation is observed in patients with advanced gout and is more frequently observed in joints with tophi [31]. Thus, osteocytes exposed to MSU crystal-induced inflammation may contribute to both aspects of disordered bone remodeling in gout: pathological bone formation and increased bone resorption.

The addition of MSU crystals to RAW264.7 cells in vitro led to increased secretion of TNF-α and PGE_2_ and, although TNF-α was secreted at higher concentrations, the downstream proinflammatory response evoked in MLO-Y4 osteocytes is unlikely to be dependent on TNF-α since neutralization of TNF-α had no major effect on the induced inflammatory response. In contrast, inhibition of COX-2 activity in MLO-Y4 cells did suppress the induced expression of IL-6, IL-11, RANKL, and PGE_2_. The molecular mechanism by which COX-2 inhibition blocked MLO-Y4 cell expression of cytokines and inflammatory mediators in response to MSU crystal-induced inflammation was not further investigated in this study. However, other studies using stromal cells, such as fibroblasts and epithelial cells, have shown a link between COX-2 activation, PGE_2_ release, and the subsequent expression of IL-6 in response to inflammatory stimuli [32, 33]. In these studies, COX-2 inhibition decreased downstream IL-6 gene and protein expression [32, 33] by inhibiting PGE_2_ activation of NF-κβ and C/EBPβ transcription factors [33]. Other research has demonstrated that addition of exogenous PGE_2_ directly induces IL-6 expression in osteoblasts and fibroblasts [24, 34]. In the current study, COX-2 inhibition did not significantly change MLO-Y4 gene expression of COX-2 but did significantly reduce MLO-Y4 production of PGE_2_, indicating that increased COX-2 activity is important for the downstream inflammatory response. In tophaceous joint samples, COX-2 protein expression was observed near sites of bone erosion. These results suggest that upregulated COX-2 activity and PGE_2_ production at sites affected by tophaceous gout may be important for driving the shift in osteocyte phenotype towards a proinflammatory and proresorptive state in response to MSU crystal-induced inflammation. COX-2 has also been implicated in bone resorption in other forms of inflammatory arthritis. In animal models of rheumatoid arthritis, COX-2 inhibition can reduce inflammatory bone erosion [35, 36]. For current gout management, COX-2 inhibitors are used as anti-inflammatory agents for treatment and prevention of gout flares. Our results raise the possibility that COX-2 inhibition may also inhibit the cellular processes contributing to pathological bone remodeling in tophaceous gout.

IL-1β plays a key role in initiation of the acute gout flare [37, 38]. In our in-vitro model of MSU crystal-induced inflammation by macrophages, IL-1β secretion was not upregulated in RAW264.7 macrophage cells cultured with MSU crystals. This is consistent with previous in-vitro studies where MSU crystals alone did not induce IL-1β release in human or murine macrophages without additional priming of cells with either lipopolysaccharide or phorbol 12-myristate 13-acetate [39, 40]. Activation of the inflammasome and release of IL-1β is critical for initiating the intense acute inflammatory response in the gout flare [37]. However, tophi are not typically acutely inflamed. Therefore, we believe that the indirect experiments using macrophage conditioned medium represent a relevant in-vitro model to examine how interactions between MSU crystals and macrophages affect osteocyte-regulated bone remodeling in tophaceous gout.

## Conclusions

In summary, MSU crystals directly reduce osteocyte viability but have no direct effects on osteocyte gene expression. In contrast, interactions between MSU crystals and macrophages indirectly promote osteocyte expression of proinflammatory mediators and factors involved in bone remodeling, particularly proresorptive factors; these effects can be suppressed with COX-2 inhibition in osteocytes. These interactions may contribute to disordered bone remodeling in tophaceous gout.

## Additional files

Additional file 1: **Figure S1.** The effect of different sizes of MSU crystals on MLO-Y4 cell viability. The alamarBlue® assay was used to determine the viability of MLO-Y4 cells cultured with different sizes of MSU crystals for 24 h. Viability was assessed 24 and 48 h after the addition of MSU crystals. Data shown are pooled from three biological repeats and are presented as mean (SEM), two-way ANOVA: *P*_Interaction_ = 0.86; *P*_MSU crystal size_ = 0.96; and *P*_MSU crystal concentration_ = 0.0001 for the 24 h time point; and *P*_Interaction_ = 0.13; *P*_MSU crystal size_ = 0.21; and *P*_MSU crystal concentration_ < 0.0001 for the 48 h time point. (JPG 154 kb)

Additional file 2: **Figure S2.** Indirect effects of MSU crystal-stimulated RAW264.7 macrophage conditioned medium on MLO-Y4 cell viability. RAW264.7 macrophages were cultured with or without 0.5 mg/mL MSU crystals for 24 h for preparation of MSU crystal-stimulated conditioned medium and control conditioned medium, respectively. Conditioned medium preparations were added to MLO-Y4 cells at different concentrations (5%, 20%, and 40% final concentration in a well) for 24 h. The alamarBlue® assay was used to determine MLO-Y4 cell viability 24 h and 48 h after the addition of conditioned medium. Data shown are pooled from three biological repeats and are presented as mean (SEM), two-way ANOVA: *P*_Interaction_ = 0.16; *P*_Time_ = 0.74; and *P*_Conditioned media concentration_ = 0.17. (JPG 152 kb)

Additional file 3: **Figure S3.** RAW264.7 macrophage expression of TNF-α and PGE_2_ in response to MSU crystals. RAW264.7 macrophages were cultured with or without 0.5 mg/mL MSU crystals for 24 h for preparation of MSU crystal-stimulated conditioned medium and control conditioned medium, respectively. The concentration of TNF-α, PGE2, IL-1β, IL-6, RANKL, and OPG in conditioned medium samples were measured by ELISA. IL-6 and RANKL were undetected in all samples. Data shown are pooled from three biological repeats and are presented as mean (SEM), two-tailed paired *t* test as indicated between groups. (JPG 156 kb)

Additional file 4: **Figure S4.** The effect of neutralizing TNF-α on MLO-Y4 cell inflammation induced by MSU crystal-stimulated RAW264.7 macrophages. RAW264.7 macrophages were cultured with or without 0.5 mg/mL MSU crystals for 24 h for preparation of MSU crystal-stimulated conditioned medium and control conditioned medium, respectively. Conditioned medium and either 5 μg/mL neutralizing TNF-α antibody or 5 μg/mL IgG isotype control were added to MLO-Y4 cells for 24 h and MLO-Y4 cells were then harvested and mRNA extracted for analysis of gene expression by real-time PCR. Data shown are pooled from four biological repeats and are presented as mean (SEM), one-way ANOVA with post-hoc Sidak’s test between groups as indicated. NS no significant difference. (JPG 203 kb)

## Abbreviations

3D: Three-dimensional
ANOVA: Analysis of variance
BCP: Basic calcium phosphate
COX-2: Cyclooxygenase-2
CPPD: Calcium pyrophosphate dehydrate
ELISA: Enzyme-linked immunosorbent assay
FBS: Fetal bovine serum
IL: Interleukin
MSU: Monosodium urate
NF-κβ: Nuclear factor kappa-B
OPG: Osteoprotegerin
PCR: Polymerase chain reaction
PGE_2_: Prostaglandin E_2_
RANKL: Receptor activator of nuclear factor kappa-B ligand
TNF: Tumor necrosis factor
