# Supplementary figures and images for: Monosodium urate crystals reduce osteocyte viability and indirectly promote a shift in osteocyte function towards a proinflammatory and proresorptive state
Source: Arthritis Res Ther. 2018 Sep 10;20:208. doi: 10.1186/s13075-018-1704-y (PMC6131786; doi:10.1186/s13075-018-1704-y)

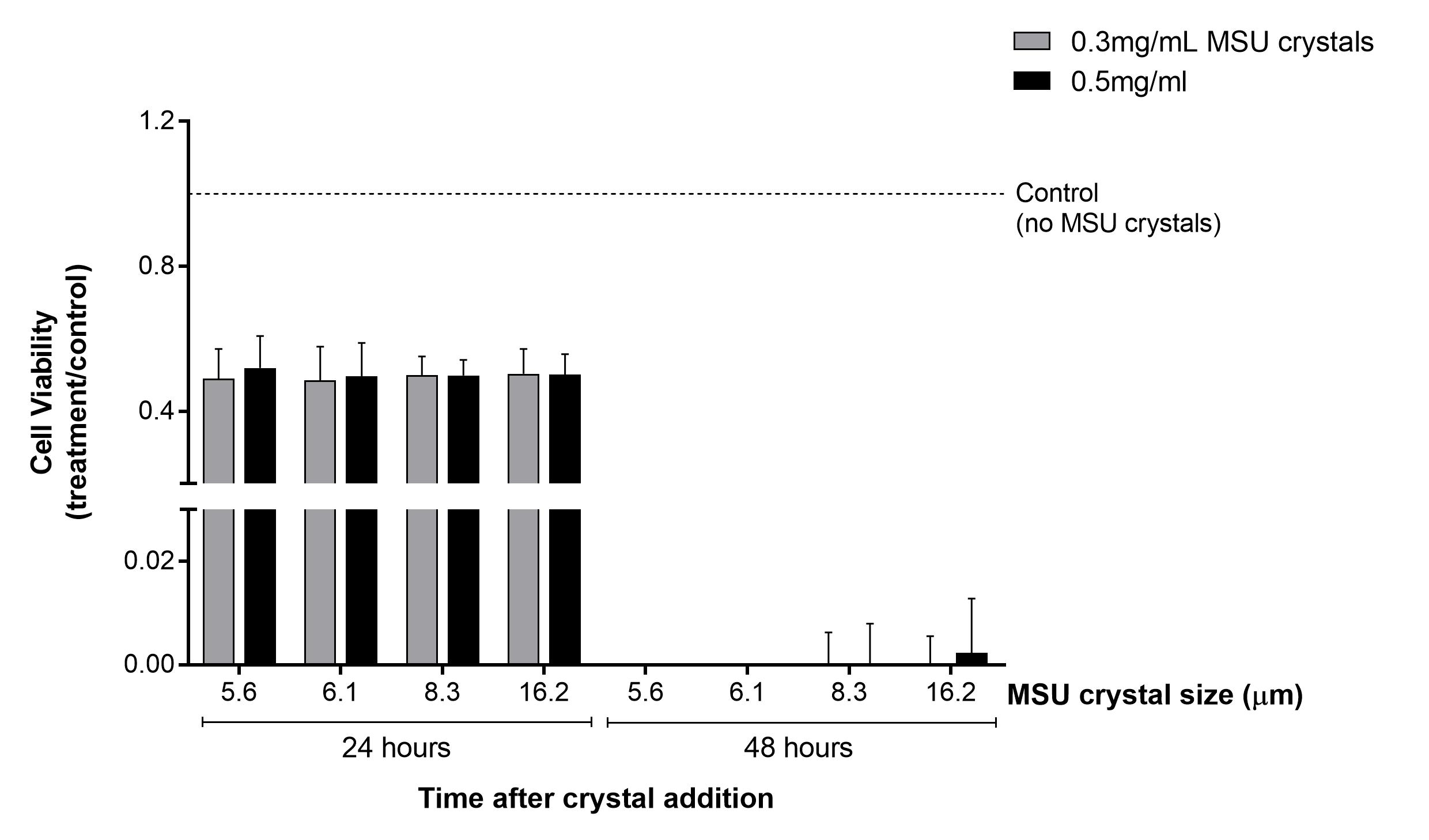

Supplement: Supplementary file 1 — Figure S1. The effect of different sizes of MSU crystals on MLO-Y4 cell viability. The alamarBlue® assay was used to determine the viability of MLO-Y4 cells cultured with different sizes of MSU crystals for 24 h. Viability was assessed 24 and 48 h after the addition of MSU crystals. Data shown are pooled from three biological repeats and are presented as mean (SEM), two-way ANOVA: PInteraction = 0.86; PMSU crystal size = 0.96; and PMSU crystal concentration = 0.0001 for the 24 h time point; and PInteraction = 0.13; PMSU crystal size = 0.21; and PMSU crystal concentration < 0.0001 for the 48 h time point. (JPG 154 kb) [file 13075_2018_1704_MOESM1_ESM.jpg]

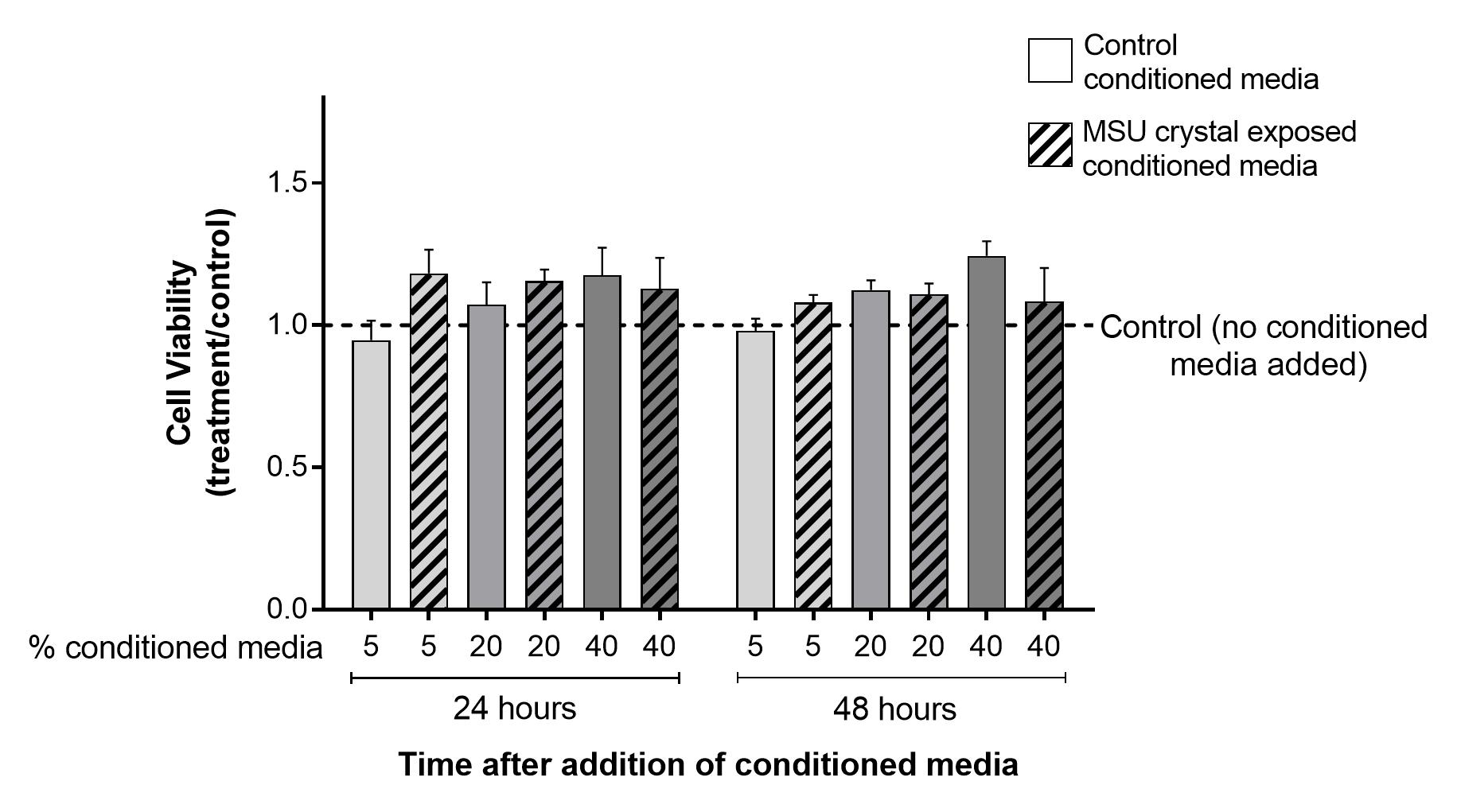

Supplement: Supplementary file 2 — Figure S2. Indirect effects of MSU crystal-stimulated RAW264.7 macrophage conditioned medium on MLO-Y4 cell viability. RAW264.7 macrophages were cultured with or without 0.5 mg/mL MSU crystals for 24 h for preparation of MSU crystal-stimulated conditioned medium and control conditioned medium, respectively. Conditioned medium preparations were added to MLO-Y4 cells at different concentrations (5%, 20%, and 40% final concentration in a well) for 24 h. The alamarBlue® assay was used to determine MLO-Y4 cell viability 24 h and 48 h after the addition of conditioned medium. Data shown are pooled from three biological repeats and are presented as mean (SEM), two-way ANOVA: PInteraction = 0.16; PTime = 0.74; and PConditioned media concentration = 0.17. (JPG 152 kb) [file 13075_2018_1704_MOESM2_ESM.jpg]

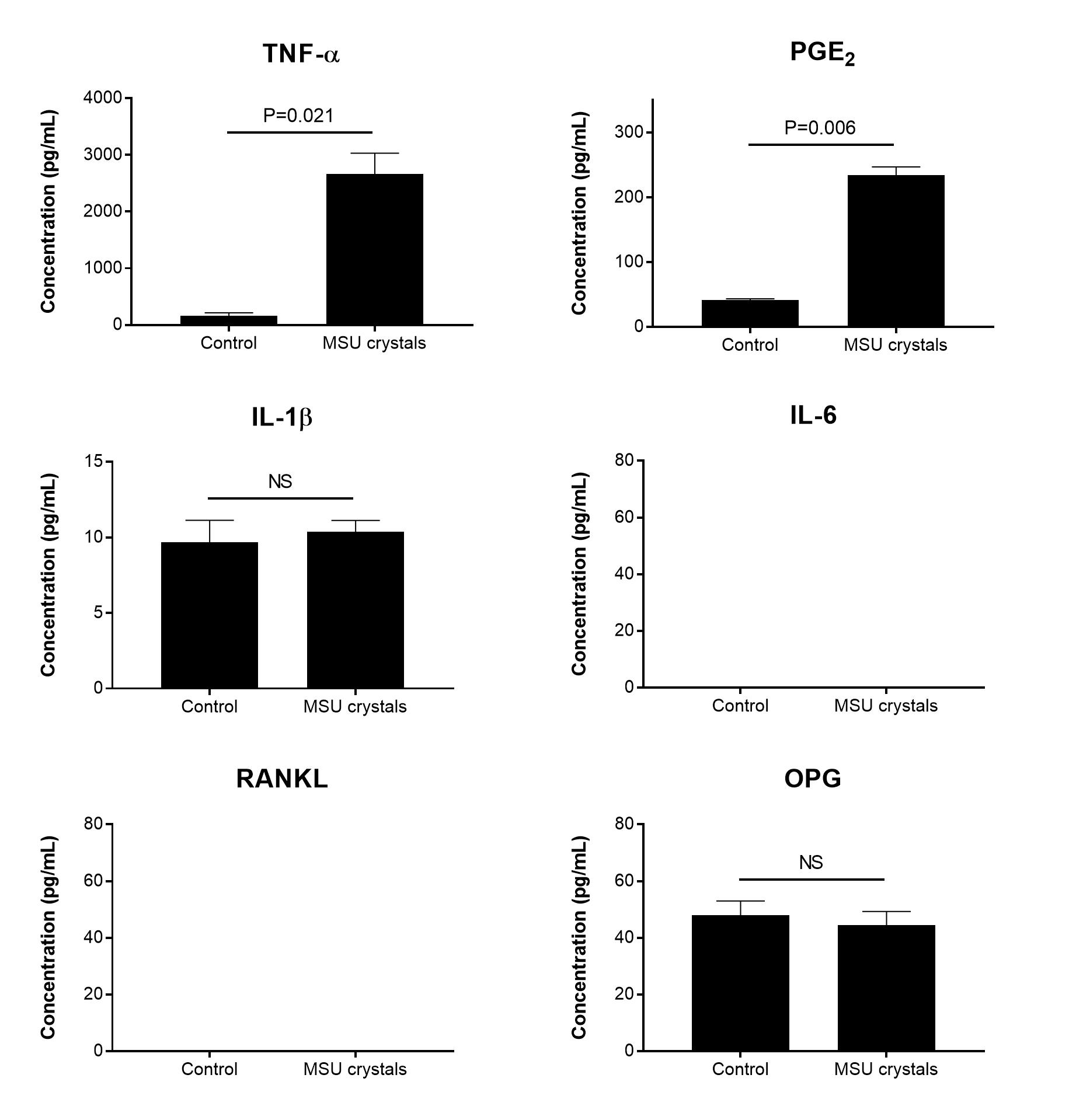

Supplement: Supplementary file 3 — Figure S3. RAW264.7 macrophage expression of TNF-α and PGE2 in response to MSU crystals. RAW264.7 macrophages were cultured with or without 0.5 mg/mL MSU crystals for 24 h for preparation of MSU crystal-stimulated conditioned medium and control conditioned medium, respectively. The concentration of TNF-α, PGE2, IL-1β, IL-6, RANKL, and OPG in conditioned medium samples were measured by ELISA. IL-6 and RANKL were undetected in all samples. Data shown are pooled from three biological repeats and are presented as mean (SEM), two-tailed paired t test as indicated between groups. (JPG 156 kb) [file 13075_2018_1704_MOESM3_ESM.jpg]

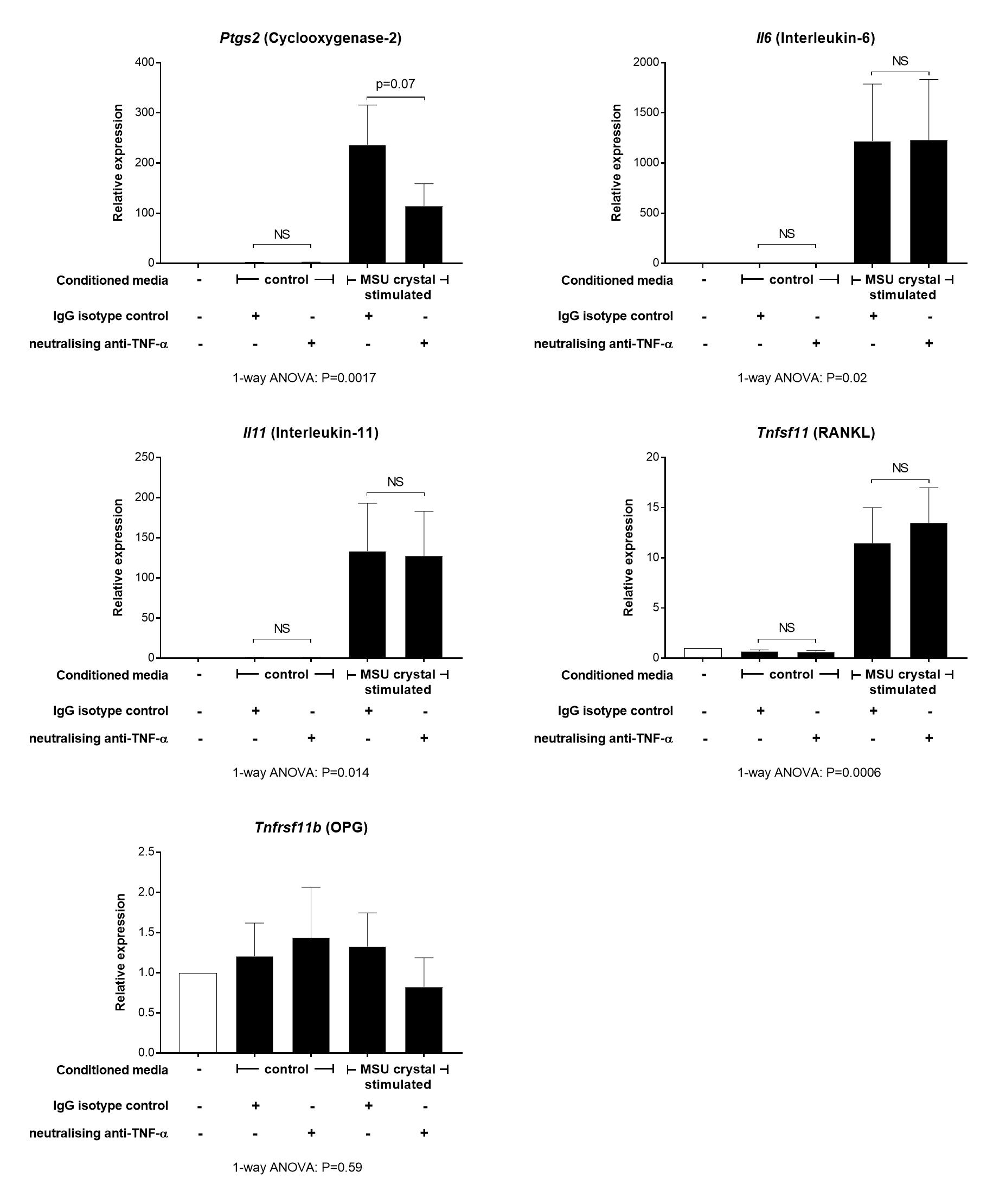

Supplement: Supplementary file 4 — Figure S4. The effect of neutralizing TNF-α on MLO-Y4 cell inflammation induced by MSU crystal-stimulated RAW264.7 macrophages. RAW264.7 macrophages were cultured with or without 0.5 mg/mL MSU crystals for 24 h for preparation of MSU crystal-stimulated conditioned medium and control conditioned medium, respectively. Conditioned medium and either 5 μg/mL neutralizing TNF-α antibody or 5 μg/mL IgG isotype control were added to MLO-Y4 cells for 24 h and MLO-Y4 cells were then harvested and mRNA extracted for analysis of gene expression by real-time PCR. Data shown are pooled from four biological repeats and are presented as mean (SEM), one-way ANOVA with post-hoc Sidak’s test between groups as indicated. NS no significant difference. (JPG 203 kb) [file 13075_2018_1704_MOESM4_ESM.jpg]
